# Sustainable development goals, universal health coverage and equity in health systems: the Orang Asli commons approach

**DOI:** 10.1017/gheg.2016.8

**Published:** 2016-07-11

**Authors:** Y. S. Wong, P. Allotey, D. D. Reidpath

**Affiliations:** 1 School of Medicine and Health Sciences, Monash University Malaysia, Petaling Jaya, Selangor, Malaysia; 2 Executive Director's Office, Malaysian Care, Kuala Lumpur, Malaysia; 3 Global Public Health and SEACO, Monash University Malaysia, Bandar Sunway, Selangor DE, Malaysia; 4 South East Asia Community Observatory (SEACO), Monash University Malaysia, Bandar Sunway, Selangor, Malaysia

**Keywords:** Indigenous Peoples, Orang Asli, universal health coverage, commons

## Abstract

Universal health coverage is a key health target in the Sustainable Development Goals (SDGs) that has the means to link equitable social and economic development. As a concept firmly based on equity, it is widely accepted at international and national levels as important for populations to attain ‘health for all’ especially for marginalised groups. However, implementing universal coverage has been fraught with challenges and the increasing privatisation of health care provision adds to the challenge because it is being implemented in a health system that rests on a property regime that promotes inequality. This paper asks the question, ‘What does an equitable health system look like?’ rather than the usual ‘How do you make the existing health system more equitable?’ Using an ethnographic approach, the authors explored via interviews, focus group discussions and participant observation a health system that uses the commons approach such as which exists with indigenous peoples and found features that helped make the system intrinsically equitable. Based on these features, the paper proposes an alternative basis to organise universal health coverage that will better ensure equity in health systems and ultimately contribute to meeting the SDGs.

## Background

A key area of health systems research has been the issue of health equity. While a health care system is commonly viewed as a complex social institution designed to provide biomedical interventions that produce better individual health, health care systems should also promote a wider set of societal values and norms that contribute to overall social good [1, 2]. Equitable access to health care for all is undoubtedly a benchmark, recognised in the Millennium Development Goals and its follow-on, the recently announced Sustainable Development Goals (SDGs). The interconnectedness of the SDGs is clear such as where healthy lives and well-being for all (Goal 3) is interlinked with ending poverty and hunger (Goals 1 & 2), reducing inequalities (Goal 10), providing clean water and sanitation facilities (Goal 6), protecting the environment (Goals 7, 13, 14, 15), providing decent work (Goal 8), ensuring gender equality (Goal 5) and having access to quality education (Goal 4). The underlying values of equity expressed is then meant to be actualised in universal health coverage (UHC) whose goal is to ensure that all people obtain the health services they need [3] and is now becoming the significant SDG health goal that links equitable social and economic development, and combines financial risk protection with equitable access to essential services [4].

There is strong rhetoric surrounding UHC and it is high on the global agenda reflected in the 2012 United Nations General Assembly Resolution [5] to ensure the highest attainable health for all. There are a number of other international instruments that attempt to provide accountability bodies that support addressing equity such as the Economic and Social Council where tackling inequality has become a major focus. For indigenous populations in particular these include the United Nations Declaration on the Rights of Indigenous Peoples, the United Nations Permanent Forum on Indigenous Issues and the United Nations Inter-Agency Support Group on Indigenous Issues. However, the good intentions are subject to several intermediary drivers that have an impact on implemention. For instance, there have been concerns raised about the lack of participation by and deliberation with local communities in determining health goals and services. Furthermore, there are major tensions as a result of the growing neoliberal dominance, which has fuelled an almost parallel discourse on privatisation and commercialisation of public goods such as health care. Indeed, the privatisation agenda is itself reflected in the SDGs on industrialisation (Goal 9). The fundamental challenge is in reconciling a system based on maximising profits with a health system that aims to achieve equity [6, 7]. In the current environment, efforts to implement UHC rest on top of a system that intrinsically promotes inequality [8]. What effect will this have on achieving UHC and further on, the ultimate success of the SDG goals?

This tension is evident in the ongoing challenges to enhance health equity among the Orang Asli, the indigenous minority population of Peninsular Malaysia. In this paper, we review some of these tensions and present the findings of a study with the Orang Asli, the aim of which is to explore what an equitable health care system looks like.

## Methods

The study took an ethnographic approach to gain an understanding of health and health care practices, which are deeply embedded in the social and cultural perspectives and norms of a society [9, 10]. The goal of ethnographic research is to understand sociocultural phenomena and ultimately, to use these understandings to bring about positive change in communities or institutions [11]. The ethnographic approach required extended and intimate face-to-face in-depth interviews to learn, understand and accurately reflect the people's viewpoints and practices in their natural setting [12, 13].

### The research setting

The study was conducted in Malaysia, an upper-middle income economy with a total population of 29 million in 2012 and a national Gini coefficient of 0.43 in the same year [14]. Since independence in 1957, the Malaysian health care system has changed from a largely national health service to one with a large private sector component particularly in provisioning urban, middle class locations [15]. For rural or poorer populations, state provided health services remain the place of choice for medical treatment but for geographically isolated populations such as many indigenous communities in West and East Malaysia, state health services is often the only choice for modern health services where health authorities periodically dispatch mobile medical clinics [16].

### Study population characteristics – the Semai Orang Asli

The Orang Asli are the indigenous peoples group of Peninsular Malaysia with a population of 178 197 [17] or just over 0.6% of the country's population. Orang Asli which means original people is a collective term referring to three ethnic groups – the Negrito, Senoi and Aboriginal Malay – who are further officially grouped into 18 different language sub-groups by the state. The Semai are one of the six language sub-groups of the Senoi inhabiting the central and southern parts of the state of Perak and the northwestern parts of the state of Pahang in Malaysia. They are also the largest in number making up just over half of the total Orang Asli population. The Senoi come from the Mon Khmer family which is distinct from the dominant Malay population which is Austronesian in ancestry [18].

The Semai are not strictly hunter–gatherers although they do engage in hunting and gathering. Anthropologists have grouped them nearer to being horticulturist, swiddeners and traders [19–21]. This distinction is important because first it shows there is heterogeneity among the Orang Asli and second, the resulting variation in how resources are held and owned is foundational to their health care system. Among hunter–gatherers, Endicott's [22] study of the Batek, another Orang Asli group, show an absence of individual or even communal ownership to land or its natural resources. And while there is close affinity to a specific bounded area of land, the idea of excluding others from living or working on it is alien. This suggests that a more open access regime operates for land and natural resources among the Batek. However, a nascent concept of exclusive communal ownership does exist whereby Batek from other areas are not welcomed to another's fruit orchard's pickings. Endicott does note that studies of other groups of Orang Asli hunter–gatherers [23] show greater propensity towards communal property rights as with the Semai and he postulates that this due to greater population pressure.

### Sampling and data collection

A total of five Semai villages in Perak were selected for sampling sites. These were chosen for their relative degree of rurality and development in relation to modern health care services. Two villages were in remote, rural areas and difficult to access due to poor road conditions while three others were located nearer urban areas accessible via sealed roads. Villages were purposively sampled to examine the possible effects of urbanisation and proximity to health services on the traditional commons health care system [24] and the usage of modern health care services.

Given this setting to obtain information on people, culture, land and environment, a number of approaches were used to facilitate triangulation of the data. Those included participant observation, in-depth interviews and focus group discussions. Data collection was conducted with the Semai community in the setting of their own villages. Six focus groups discussions (FGDs) and a number of in-depth interviews were held with a total number of 76 participants; with between 10 and 20 people attending each FGD. The numbers for each were difficult to control in spite of prior organisation. The variation in attendance at the FGDs arose because of the open, fluid and participative nature of village discussions. Special invitations and arrangements were made to the older members of the community to participate as knowledge about traditional systems resided more with them; nobody was excluded from participation.

The lead researcher (Wong) has a longstanding relationship with the community as a director of a non-government organisation that has worked alongside the community in poverty eradication initiatives. FGDs were preceded by lengthy interactions covering the latest news from outside the community, as well as local social events. This process helped to reinforce the relationship between the researcher and community. FGDs would then begin with an introduction covering the purpose of the discussion and the study, and the ethical guidelines ensuring that discussions were held under Chatham house rules [25]. Discussion topics across the different groups followed a guide, however, some flexibility was maintained to allow the participants to direct the level of depth and details of the various topics. The participants therefore spoke at length about the traditional health care system and issues related to the modern health care system and the effects of changing social conditions.

Field notes were recorded during unstructured interviews with various members of the community and over long periods when the lead researcher was with the community observing their usage and care of land and natural resources, their interactions and relationships with each other and their interactions with outsiders. Data from FGDs were audio recorded and transcribed by the researcher. The translations were verified by an Orang Asli assistant to confirm accuracy where discussions were conducted in the Semai language. Transcribed text was imported into nVivo [26] software and the data analysed using thematic analysis, so that we were faithful to the context and its complexity but at the same time rendering it meaningful for wider issues [27].

The broad themes identified related to the workings of the traditional health care system focusing on how land and natural resources were communally held and used, social protection and various types of medicinal plants and treatments. Participants discussed rules that governed who could use those resources and how development was affecting the resource base on which their traditional health care depended on. A second set of themes related to the participants’ experiences with the modern health care system and its effects on the traditional system. Participants shared about how they understood the causes of illness and treatment as informed by state health care workers and how they coincided or diverged with their traditional views. A third set of themes centred on how indigenous knowledge was communally owned and how it was being transmitted or lost to the new generation of Orang Asli youths. Lastly, a set of themes emerged on their experiences and views of marginalisation in relation to health, health care services and development.

## Results

### Indigenous peoples and health care systems

Indigenous peoples traditional health care practices have been the subject of much interest and study from a local to international level [28]. Most of the interest centred around an anthropological interest in individual ethnic group's practices, or botanically, of the taxonomy and usage of medicinal plants. Here we will take a systems approach instead. In debates over its relevance to modern health care, indigenous people's health care practice is often relegated to the periphery and within a biomedical system regarded as irrational or simply wrong [29]. From an emic perspective, however, indigenous health care practices are embedded in an elaborate and coherent system that meets the key needs of its people, their community and the environment.

In describing their health system, the key concepts centred around the notion of shared commons and property rights to resources integral to the maintenance of individual health and the well-being of the community. The commons or common property regime is a social construct where a socially recognised group of people have collective or communal ownership of and exclusive use rights to a property. Individuals accepted as members into the group exercise usufruct rights in conjunction with reciprocal obligations to the accepted perimeters of usage. A property can be cultural or natural resources that are shared by all members of a community and in traditional societies these may include shared lands, water sources, public property or social protection systems [30, 31].

Most literature on the commons has revolved around natural resources [30–35] and knowledge [36–38], but a handful of writers have begun to analyse health care using a commons perspective. Smith-Nonini and Bell [39] use this perspective to analyse tax-based and market-based national health care systems, while Lewis [40] looks at the sustainability of a health care service if it operates as a common pool resource.

Wong *et al*. [24] went further to conceptualise indigenous peoples traditional health care system as a commons health care system that features communal rights and responsibilities to natural resource usage, knowledge transmission and social protection. This arose as a result of an egalitarian social structure, the wide diffusion of power and the common property ownership regime found in most indigenous peoples societies. For them, the most important property is land and natural resources such as forest. From it comes their food, water, medicinal plants, energy and building materials for shelter foundational to maintaining physical health and livelihoods [41–44]. Many indigenous peoples have developed land and forest usage practices that protect and preserve the environment for its ecosystem functions such as erosion control and climate regulation that are essential to human health [45–47]. The land and forest forms their social and spiritual backdrop providing a community with their belief system, their individual and ethnic identity as a unique group, and their cultural practices, each contributing to the self-worth and social cohesion of the community [48, 49]. Colfer describes the negative impacts of the loss of such socio-cultural settings experienced by communities traditionally linked with their land and forest environments.

### Communal ownership of land and natural resources – rules of engagement

The Semai too have a close affinity to the land and forest. They have a strong economic investment in horticulture and swiddens that require periodic tendingand so accordingly, they have a definitive way to manage the use of its resources.

Like many other indigenous peoples groups, the Semai concept of ownership of land and natural resources is as a collective. However, common property ownership did not mean open access to anyone, as was frequently misunderstood because that leads to unsustainable use of resources [32]; it is definable and exclusive to group members with a strong shared identity. The participants in the FGD at Kampung Ulu Rasau described how territoriality within communal ownership was practiced:

We who are from Rasau definitely are of Rasau; for example from the river mouth to its head waters. Then as the trees are growing big, we shift in a circle and the graves are here (in the middle) until we come back to the start. This is what we mean when we say we are people of Rasau. For example in Pahang at the Pahang river. The tributaries near the Pahang river where there is a village. Don't you go and look for fish there. Cannot! You cannot because the village area follows the tributaries of the river. That was how it was.

By controlling access, territoriality ensured that resources were not overextracted to the detriment of the well-being of a resident group.

Communal ownership of land and natural resources meant members of the same group had equal access to these resources in their territory. As long as a group member made an effort to work the land or gather produce and had the necessary knowledge to do so, then he or she was free to access and utilise the resources within their territory [50]. The rule for Semai from other groups who wished to gather food or medicinal plants was that they had to first obtain permission from the resident group before they could take out materials. In an interview, this farmer who has his own oil palm plantation from Kampung Bota even likens it to a government policy:

*Yes, ask. Like there was once someone wanted to go into the forest to get some leaves and roots to cure his body, he discussed with my grandfather first like there is a condition. This is for outsiders. It is like there is a bit of a policy*.

*Maybe different places have their own different ways. Each has their conditions*.

*As the saying goes different field, different grasshopper*.

The rules become more stringent with non-Semai people and the following accounts from a FGD in Kampung Ganggai suggests that past collective experience with other people groups were an influencing factor:

This is the situation; if it is with the Malays, its better they don't know about our medicines because they will take everything and then where will we find our medicines?

*What the headman means is that between Malays and Chinese, the Chinese will take little, because they will take what they need and that's it. Malays will take bit by bit, one, two, three and before long all is gone. Our nenqriiq (ancestral domain/territory) will be finish*.

Definable boundaries and rules that match local needs are two key design principles that contribute to a successful and sustainable common property regime [35] and here we see that the Semai have established in their system boundaries to their land and who can access its resources including medicinal resources.

Within these rules of engagement, the Semai traditional health care system: (1) guarantees equal access to food, shelter and medicinal resources for resident group members; (2) allows Semai from other groups to access these resources once permission is given by the resident group; and (3) restricts the entry or extractive prospect of non-Semai based on their track record with the resident group. Together, these helped to ensure for the Semai equality in access and sustainability of their resources so as to not jeopardise the well-being of a community.

### Reciprocity and social obligation

In a commons health care system, the benefits of ownership for group members come with obligations. Exercising property rights meant fulfilling social and cultural obligations such as taking care of the young, the old or the sick [50, 51]. It is these obligations that form the basis of another key feature of the commons health care system – the social protection base [24]. Among the Semai, these reciprocal social obligations helped ensure that group members had sufficient food, had access to medicines and traditional health treatment, and were taken care of when ill, very young or very old. The women in Kampung Bota participating in a FGD shared this:

*That is normal. When we are in difficulty then they also help and when they need help, we help in turn*.

*Yes, this is our custom of helping each other out*.

*Help. Never been a case where a person is left abandoned*.

For the Semai, practicing these social obligations arose first from compassion towards fellow group members in need of help and second, awareness of the risks of sanctions expressed in forms of social or cultural taboos. Recalling how his grandmother used to care for them, an elderly man from Kampung Ulu Rasau had this to say:

*Like with meat if it is a big piece, then she will give too. The way she will apportion is like with the ribs she will apportion equally among all families even if there are four or forty households. The legs, the ears, all will get a share*.

*Not one will be left out*.

If they apportion wrongly and someone doesn't get their share, in a day or two there will be a village discussion. Cannot!

*We call it ‘penali’. It is like if you don't follow the practice it will be inauspicious*.

*It is because if they didn't do that, it showed they had no love. Second, it means they will be damned – punishment for breaking rules*.

Together, compassion and the risks of sanctions helped motivate the collective role of group members to maintain good social relations and the overall well-being of the group and its environment.

### Local and small group settings

The ability for social obligations, sanctions, good relations and communal ownership to be applied effectively require a level of intimacy that was only feasible in localised and smaller group sizes. The Semai lived in relatively small communities that traditionally ranged from 5 to 15 related households. The larger settlements of over 100 households with disconnected dwellings evident today are mainly due to regroupment and resettlement schemes enforced by the state. In the regroupment scheme of Pos Tenau, the people participating in a FGD shared this:

*It is because in the past the whole village stayed in one big house so we could see everyone and take care of each other. Not like now we stay in separate houses one by one*.

*My father's family together with his brothers and sisters would build one big house. One big house with many rooms. But the guest space is definitely big*.

*Whoever is sick in the house everyone will help to take care, everyone keeps watch*.

Local and small community groups provided a more effective setting for the commons health care system to operate because people knew each other better and could watch out for one another.

### Communal ownership of knowledge

For the Semai, knowledge is held as collective knowledge that is shared not only among an existing group but spans generations handed down in oral form and through repeated practice. As with many indigenous groups, it is this knowledge in adaptive livelihoods, conservation, ecosystem management and appropriate governance structures that sustains and allows communities to thrive [52]. Additionally, such knowledge is not just information but is fundamental to their identity as individuals and as a community with a history [53]. This form of collective knowledge is clearly evident in matters of the health and well-being of a community in a subsistence economy. Health care knowledge has to be mutually known for reciprocity to function because if other group members do not know how to obtain and prepare medicinal plants or take care of the sick or weak, operationalising a group's social obligations becomes impossible. As such for the Semai, health care knowledge is passed on to anyone in the group who is interested irrespective of gender or age.

Like many traditional societies, Semai see health as closely linked to the spirit world and illness as the result of problems with spirits [54] or the violation of social and cultural taboos [55, 56]. Thus, the knowledge needed in traditional health care addresses the physical, socio-cultural and spiritual causes of illness. Expressed in every sampling site, knowledge is held in common, passed down orally through generations and accessible to all in a group:

*Anyone can learn so long as they are interested*.

*We know all these from the stories of our grandparents, our ancestors passed down the knowledge*.

*Passed down each generation*.

*The old people showed us the different kind of leaves and at the same time taught us how to use it. We would follow what they taught*.

*They would show which is for stomach aches, headaches, fever, diarrhea. Then those which are for curing people who are disturbed by spirits in the forest*.

What we see here with the Semai is that monopolising knowledge was unacceptable and that the sharing of knowledge was part of the social obligation of group members. This egalitarian and communal way of holding knowledge helped promote equality since there was very little knowledge not accessible to every group member thus enabling them to in turn access resources needed for their health and well-being. The modern practice of monopolising knowledge through patents and copyrights or requiring payment to share knowledge was inconceivable in a commons system.

## Summary of findings

Our findings point to an integration of features found in the commons health care system that together act to ensure equity in health care services for members of a community. Communal ownership of resources guarantees group members equal access to health treatment and resources. These resources include land for dwellings and crops, natural resources for medicines, sustenance and livelihoods, and the knowledge required to use the resources to maintain good health. This guarantee however applies with the expectation that group members fulfill their social obligations to other group members. Failure to fulfill these obligations could lead to sanctions being applied for failures to reciprocate or close supervision by the community for fear of inauspicious incidents linked to cultural taboos. These features within the limits of its technology provided the access, the treatment and the aftercare of the commons health care system.

For such undertakings to succeed, the key seems to be that they needs to operate in localised and smaller community groupings as this allows each member to know each other closely and are drawn from a set of common values and perspectives enabling them to apply effective influence. The ability to establish intimate community relations becomes more difficult in large groups making reciprocal social obligations and sanctions similarly harder to operationalise except through enforcement by law. As population sizes continue to grow, the pressure of maintaining health care services in smaller community groupings will also grow with rising demand.

The concept of sharing is not limited to material property but include knowledge [57]. Our findings indicate that the knowledge commons in the commons health care system functions as: (i) a shared repository of accumulated knowledge that is open to everyone from the group; (ii) an enabler for group members to give health treatment and to operationalise reciprocal social obligations; and (iii) an equalising mechanism that enables women and younger people to participate in decision making. This does not mean that there was no difference in capabilities between different ages or gender because acquiring the knowledge depended on time, interest and diligence. It also does not mean that individuals or groups of individuals do not have specialised knowledge such as a traditional healer would but the knowledge was freely available to anyone in the group if they wanted to learn. The more time and diligence invested in acquiring the knowledge determined the person's capability.

Interestingly, modern day society is beginning to rediscover the importance of a knowledge commons in an age dominated by proprietary knowledge and this has largely been made possible by the internet [37].

## Discussion

This exploration of equity and health care systems asks the question what an equitable health care system looks like. With growing inequalities in the world, it is a particularly pertinent question because current health care systems are locked into an increasingly privatised regime or overtaxed in the state owned regime. Tackling this equity issue will need more than just tweaking the existing health care systems but some fundamental rethink. While indigenous peoples in developed countries such as Australia, Canada or New Zealand have made encouraging progress in engaging with mainstream health systems and giving some legitimacy and standing to traditional health systems in what are often referred to as intercultural health services [28, 58, 59], this is not necessarily reflected with indigenous populations in low and middle-income countries such as Malaysia. The traditional health care practiced by the Semai remain a quaint practice of collecting herbs from the back garden blown over from a bygone age. To the Orang Asli, however, it continues to provide essential health care. In addition, when shown to be a coherent health care system, its equity promoting values and features parallel the underlying values of UHC and can improve its implementation by reviewing:

1. Patterns of participation and ownership of health care services

Key to success is the participation of stakeholder communities in the system. Greater community participation by citizens or the community in health care services targeted for them is widely regarded as important [60, 61] because it better addresses local needs [62–64], it promotes a sense of societal goodness and social well-being [2], and leads to more equitable power relationship between the providers and recipients of services [65].

One weakness with the rhetoric of participation in the field of development is that they are still predicated on the fact that the ‘centre’ often decides how far participation happens with the ‘periphery’ and this can result in mere tokenism [66–70] proposes that for community participation to be effective, it must be an integral part of a community's experience. The commons health care system does this by proposing that beyond the benefits or gaps of a participatory approach, communal ownership in a commons health care system helps cement equity into services and resource allocation because it is an intrinsic part of the system rather than merely an intervention introduced by outsiders. Local communities should have co-ownership of local health care services that is legally recognised and these local services could be clustered to provide ownership representation at district and national levels in order for policy making at higher levels be influenced. Gavin Mooney [71] postulated about the need for a ‘constitution’ where the values and preferences of local communities were elicited and used to guide decision-making of policy makers and public officials in order for health services to be inclusive of local needs. Having local communities co-own services would go a step further to ensure that community values and preferences do not just guide decision-making but have a measure of authority to see to its implementation.

1. Health administration into localised and smaller groupings

The importance of trustworthy relationships between health provider, patients and communities is crucial for co-ownership and mutual aid to function as it provides a safe environment where accurate information and proper health treatment can be exchanged. This is true for the human health provider such as doctors or nurses as well as for an organisational system where waiting times or continuity in care contribute to or undermine such relationships [72]. In the modern health care sector, small localised groupings such as through the family physician, the general practitioner or the local primary care centre had traditionally been the environment where close relationships with patients and communities were fostered.

This has been compromised by the incessant drive towards perceived efficiencies of a market-driven system. Doctor–patient relationships have increasingly come under pressure when neo-liberal policies are translated into the health care sector. For example, a Commonwealth survey found that in managed care contracts where incentives were provided for the greatest number of patients with reduced use of resources, physicians and patients reported a decline in satisfaction due to time-related pressures [73]. Studies indicate that longer time spent with patients mean physicians are able to obtain more accurate information of a patient's health conditions [74–76], have a greater likelihood of detecting psychosocial health conditions [77] and are able to do more in terms of health education [74, 78].

Reviving these relationships means a return to small localised groupings with a people-centred environment is an essential requisite for an equity-based system to succeed.

1. Approaches to transmitting health knowledge as a commons

Access to health knowledge is increasingly being challenged. This comes firstly from the emphasis on the commercialisation of knowledge by private industries and the restriction of information by the state that is resulting in the enclosure of the knowledge commons [79–83]. Inspired by and modelled after collective action in commons based systems, some libraries, universities, non-profit publishers and professional societies collaborate to counter this new form of enclosure and produce, preserve, share, disseminate and act as repositories for information to ensure there is equitable access [84].

Secondly, the increasing complexity of medical knowledge and health training is putting it beyond the reach of people especially marginalised groups. This presents a huge challenge because the mastering of such complex knowledge requires extensive and expensive training only a select few would or could pursue. A possible solution is to this is via the use of information technology and the internet to make it accessible to people. Computerised guidance and decision support is now ubiquitous in the developed world allowing people who have no formal medical training to be knowledgeable about health and disease, and participate in decision-making over their own health with health providers [85]. The challenge now is to make it available and accessible to populations’ particularly marginalised groups such as indigenous peoples where access to basic education and information technology is often as poor as access to basic health care. This again reinforces the case about the interconnectedness of addressing health, education, poverty and economic development in the SDGs.

## Conclusion

The concept of UHC plays a fundamentally important role in promoting equitable access to health. Endorsed by WHO since 2005, it is now primed for a leading role in meeting SDG targets [86]. While there is consensus on its importance, there seems to be little consensus on anything else about it [87]. Since the gap between the concept of UHC and operationalising it depends on how it is defined, the current global emphasis is clearly narrowed down to health care financing followed by clinical health services [88]. This is not surprising given that the implementation of UHC is not independent of the health system it is in. In an era of privatisation, private health care services have increasingly replaced public services as the main provider of health care to populations and predictably, its emphasis has been in financing, and biomedical interventions and research [89].

Privatisation in health care is flourishing in the wake of neoliberal dominance in the economic and political structures of global and national governance [89, 90]. It is a global trajectory that is matched by developments in the Malaysian health care sector over the past 30 years where the Orang Asli find themselves [15]. Continuing to implement UHC in a health system that is based on a political and economic system that intrinsically promotes inequality will inevitably jeopardise the SDG goal to achieve health equity.

On the other hand, the commons health care system displayed by indigenous peoples in this study of the Semai offers a look at a system that promotes equality. It is grounded in the concept of a shared commons that has key features important to ensure equitable access to health resources and promotes reciprocal obligations that provide social protection for the sick and weak in a community. We argue that the commons health care system is conceptually far closer to UHC than the dominant private health system and with its features provides a practical basis from which UHC can be better implemented.

Ultimately, the inclusion of marginalised communities in decision making, ownership and implementation of UHC will be critical if it is to really benefit those whom universal coverage is primarily meant for. The statements on the right to health of indigenous peoples should be reflected not only in the multitude of existing declarations and mechanisms at the international level but right through into district and local governance levels particularly in less developed and developing countries. Without this, UHC will end up disproportionately benefitting dominant and wealthier groups [87, 88] rather than marginalised communities like indigenous peoples and the promise of the SDGs will remain rhetorical.
